# What's hampering measurement invariance: detecting non-invariant items using clusterwise simultaneous component analysis

**DOI:** 10.3389/fpsyg.2014.00604

**Published:** 2014-06-20

**Authors:** Kim De Roover, Marieke E. Timmerman, Jozefien De Leersnyder, Batja Mesquita, Eva Ceulemans

**Affiliations:** ^1^Methods, Individual and Cultural Differences, Affect and Social Behavior, KU LeuvenLeuven, Belgium; ^2^Heymans Institute of Psychology, University of GroningenGroningen, Netherlands

**Keywords:** measurement bias, configural invariance, weak invariance, metric invariance

## Abstract

The issue of measurement invariance is ubiquitous in the behavioral sciences nowadays as more and more studies yield multivariate multigroup data. When measurement invariance cannot be established across groups, this is often due to different loadings on only a few items. Within the multigroup CFA framework, methods have been proposed to trace such non-invariant items, but these methods have some disadvantages in that they require researchers to run a multitude of analyses and in that they imply assumptions that are often questionable. In this paper, we propose an alternative strategy which builds on clusterwise simultaneous component analysis (SCA). Clusterwise SCA, being an exploratory technique, assigns the groups under study to a few clusters based on differences and similarities in the component structure of the items, and thus based on the covariance matrices. Non-invariant items can then be traced by comparing the cluster-specific component loadings via congruence coefficients, which is far more parsimonious than comparing the component structure of all separate groups. In this paper we present a heuristic for this procedure. Afterwards, one can return to the multigroup CFA framework and check whether removing the non-invariant items or removing some of the equality restrictions for these items, yields satisfactory invariance test results. An empirical application concerning cross-cultural emotion data is used to demonstrate that this novel approach is useful and can co-exist with the traditional CFA approaches.

## Introduction

To assess the quality of psychological instruments (e.g., surveys, questionnaires, etc.), confirmatory factor analysis (CFA; Lawley and Maxwell, 1962) is often applied. CFA tests whether or not a particular latent variable model, specifying which latent variables (i.e., factors) are measured by which items, complies with the observed item scores. When the instrument is used among several groups, quality testing becomes more intricate, as the equality of different aspects of the latent variable model has to be verified (i.e., the configuration and size of the loadings of the items on the factors, item intercepts, unique variances), before the factor scores of the different groups can be compared meaningfully. For instance, when investigating cross-cultural differences in emotional experience, one has to make sure that the items of the emotion questionnaire behave the same across cultural groups. The different tests involved pertain to different levels of measurement invariance (Meredith, 1993; Meredith and Teresi, 2006) and can be performed using multigroup CFA (Jöreskog, 1971; Sörbom, 1974). In this paper, we propose a new procedure to detect which items violate configural and/or weak measurement invariance. Thus, we focus on equality of within-group covariance structures and do not consider invariance of intercepts or unique variances, or structural invariance (i.e., invariance of factor means, variances, and covariances). The novel procedure is rooted in component analysis^1^ and circumvents some disadvantages of the existing solutions in the multigroup CFA framework.

Configural invariance, which usually is the baseline model in invariance testing, implies that the same number of factors and the same pattern of zero and free loadings is imposed in all groups. The configural invariance test examines whether the items are associated with the same factors in all groups or, in other words, whether the same latent variables are measured across the groups. Weak invariance (also referred to as “metric invariance”) additionally investigates between-group agreement in how these latent variables are manifested. Specifically, it tests whether all factor loadings are equal across groups.

Traditionally, measurement invariance testing relied on conducting likelihood ratio tests (LRT) to evaluate whether adding invariance constraints caused a significant difference in the χ^2^ fit statistics. This approach has two drawbacks, however. First, its performance heavily depends on sample size (Brannick, 1995; Kelloway, 1995). Second, in large samples even tiny violations, that are not interesting from a substantive point of view, result in a rejection of measurement invariance (Note that this is exactly what a hypothesis test ought to do). To circumvent the two drawbacks associated with LRT testing, alternative goodness-of-fit indices, such as the comparative fit index (CFI; Bentler, 1990) and the root mean square error of approximation (RMSEA; Steiger, 1989), have been developed. Criteria have been proposed for deciding whether these fit indices indicate good fit (Bentler, 1990; Hu and Bentler, 1999; Tabachnick and Fidell, 2005) and whether changes in these fit indices are meaningful or “practically significant” in the context of measurement invariance (Cheung and Rensvold, 2002). Throughout this paper, following Cheung and Rensvold (2002), we will use the CFI and consider a multigroup CFA model to have a good fit when the CFI is larger than 0.95 and a more constrained model to have a “significantly” worse fit than a less constrained model when the difference in CFI (ΔCFI) is larger than 0.01.

When configural and/or weak invariance cannot be established, different latent variables appear to be measured across the groups (i.e., no configural invariance) or the same latent variables are measured differently in these groups (i.e., no weak invariance), implying that factor scores cannot be sensibly compared across groups (note that the computation of factor scores has been vastly debated; e.g., Green, 1976; Gorsuch, 1983; Grice, 2001). In the multigroup CFA framework some solutions to this problem have been proposed, which aim at detecting which restrictions on the factor loadings should be removed.

A popular strategy ^2^ is the sequential model modification procedure (MacCallum, 1986; MacCallum et al., 1992), which uses modification indices to assess whether in specific groups secondary loadings are needed for some items (to solve the lack of configural invariance) and/or to detect which loadings should be allowed to vary across groups in the weak invariance model (leading to partial weak invariance; Byrne et al., 1989); such modifications are implemented one by one. A disadvantage of this method is that in each step of the procedure, the calculation of the modification indices is based on the assumption that all other loadings (except for the ones that were deemed to be non-invariant in the previous modification steps) are invariant. When this is not the case, the modification indices are inaccurate and may lead to incorrect modifications (Williams and Thomson, 1986; Cheung and Rensvold, 1999). Also, progressively modifying the factor model until it fits the data of all groups, increases the risk of capitalization on chance (MacCallum et al., 1992; Stuive et al., 2009).

Another strategy for dealing with violations of weak measurement invariance, is item-level invariance testing (Cheung and Rensvold, 1999). Assuming configural invariance, this method first checks whether some of the factors are non-invariant with respect to their loadings. Next, it examines for each of the *n* non-zero loadings on a non-invariant factor whether or not it can be restricted to be equal across groups. This entails conducting *n*(*n* − 1)/2 invariance tests (i.e., one for each non-redundant combination of an invariant item and a reference item^3^) per non-invariant factor and integrating the results of these tests by means of a “triangle” heuristic. Specifically, an item is considered to be invariant with respect to the factor in question if restricting its loading to be equal across groups yields a CFI decrease smaller than 0.01, whichever of the other invariant items is used as a reference item (for more details, see Cheung and Rensvold, 1999).

Finally, Byrne and van de Vijver (2010) propose to delete all items one by one and to re-evaluate each time the goodness-of-fit of the multigroup CFA model. An item is flagged as non-invariant when its deletion causes the CFI to increase more than 0.01.

All three strategies become cumbersome if the number of items grows larger, because they are prone to chance-capitalization and are computationally demanding, and because their validity stands or falls with the validity of some stringent assumptions. Hence, although CFA solutions exist and are often used, these solutions are not without problems.

In this paper, we propose an alternative procedure for detecting items that are non-invariant with respect to the structure or size of their factor loadings. Our procedure circumvents some disadvantages of the CFA solutions in that it is fast and does not entail assumptions with respect to the invariance of certain items or loadings. It builds on the results of a clusterwise simultaneous component analysis (SCA; De Roover et al., 2012). Being an exploratory technique, clusterwise SCA assigns the groups under study to a few clusters based on differences and similarities in the component structure and thus in the covariance matrices of the items. Next, non-invariant items can be traced by comparing the cluster-specific component loadings (which is far more parsimonious than comparing the component structure of all separate groups). To do this in a consistent way, we present a heuristic that is based on the Tucker's congruence coefficient (Tucker, 1951), an index that is often used in, amongst others, cross-cultural psychology, to make statements about the similarity of group-specific factor structures (Lorenzo-Seva and ten Berge, 2006). Afterwards, one can return to the multigroup CFA framework and check whether removing the non-invariant items or removing some of the equality restrictions for these items, yields satisfactory invariance test results.

Clustering the groups based on their component structure is a unique feature of our approach, that makes it especially appealing when the number of groups is large. Indeed, in such cases the clustering parsimoniously reveals the most important structural differences whereas the CFA solutions discussed above quickly become very tedious and impractical. Vice versa, when the data comprise only a few groups, it makes less sense to cluster the groups and the traditional approaches may be preferred.

The remainder of this paper is organized into three sections: in the Methods section, we introduce some notation regarding the data and discuss preprocessing. Next, we recapitulate clusterwise SCA and present the heuristic for the detection of non-invariant items. Then, the Applications section illustrates the procedure using an empirical data set from research on emotional acculturation including emotional patterns from 13 different cultural groups. Finally, the Discussion will address some limitations and strengths of the presented method as well as directions for future research.

## Methods

### Data

In this paper we will be working with multivariate multigroup data, consisting of a *N*_*k*_ (subjects) × *J* (items) data matrix **X**_*k*_ (*k* = 1, …, *K*) for each of the *K* groups under study. Since clusterwise SCA aims to cluster the groups based on the within-group component structure and not on differences in group-specific item means, it is essential that the data of each group are centered per item. Moreover, since items with a higher amount of variance may dominate the obtained components, it will often be wise to rescale the data to eliminate differences between the items in measurement scale or variability^4^. As configural and weak invariance pertain to the covariance structures of the groups, we advocate to normalize the items over all groups, implying that (co)variance differences among the groups are retained in the data. That is, we recommend to analyze the **X**_*k*_ matrices, computed from the raw (i.e., unpreprocessed) data matrices **X**^r^_*k*_ as follows:

(1) $$X_{k}=(X_{k}^{\text{r}}-1_{N_{k}}{\bar{x}}_{k})S^{-1}$$

where **1**_*k*_ is a *K* × 1 vector of ones, **x**_*k*_ is a 1 × *J* vector containing the group-specific item means, **S** is a diagonal matrix containing the standard deviations of the items over all groups.

### Clusterwise SCA-P

Simultaneous component analysis (SCA; Kiers and ten Berge, 1994; Timmerman and Kiers, 2003) reduces the data of all groups simultaneously, summarizing the observed items by means of a few components according to the item covariances. SCA assumes that the same components underlie the data of the different groups and thus that the same loading matrix can be used for all groups. Specifically, the SCA model is given by:

(2) $$X_{k}=F_{k}B^{\prime }+E_{k}$$

where **F**_*k*_ (*N*_*k*_ × *Q*) denotes the component score matrix of the *k*-th group, **B** (*J* × *Q*) denotes the loading matrix which is identical for all groups and therefore does not have an index *k*, and **E**_*k*_ (*N*_*k*_ × *J*) denotes the matrix of residuals. In SCA-P, the most general variant, the variances of the component scores over all groups are fixed at one. This restriction only partly identifies the solution, in that the components of an SCA solution can be freely rotated without altering the fit of the solution. In SCA-P, the variances of and the correlations between the retrieved components may vary across the groups. Consequently, it may occur that a specific component has little variance within particular groups, or that two components have a very high correlation for one group and almost no correlation for the other groups. Apart from that, SCA-P leaves no room to find differences in covariance structure between groups.

To more extensively trace between-group differences and similarities in the component structure, clusterwise SCA (De Roover et al., 2012) was developed. Clusterwise SCA partitions the *K* groups into *C* clusters and models the data of the groups within each cluster with a simultaneous component model. In this paper, we will use the most general clusterwise SCA variant, i.e., clusterwise SCA-P (De Roover et al., 2013b), which applies SCA-P (see above) within each cluster. In this paper, given that we assume that each group is characterized by the same latent factor structure (apart from differences in the factor variances and covariances), we restrict the number of components *Q* of the cluster-specific SCA-P models to be the same across the clusters (for other purposes, clusterwise SCA extensions exist that allow the numbers of components to vary across clusters; see De Roover et al., 2013a).

Formally, clusterwise SCA-P models the data of one group as follows:

(3) $$X_{k}=\sum_{c\;=\;1}^{C}p_{kc}F_{k}B^{{\left(c\right)}^{\prime }}+E_{k}$$

where *p*_*kc*_ denotes the entries of the binary partition matrix **P** (*K* × *C*) which equal 1 when group *k* is assigned to cluster *c* and 0 otherwise and **B**^(*c*)^ (*J* × *Q*) is the loading matrix of cluster *c* (*c* = 1, …, *C*). Given that the SCA-P models per cluster are independent of one another, the cluster-specific components can be freely rotated within each cluster.

To fit a clusterwise SCA-P solution with *C* clusters and *Q* components to a given data set, the sum of the squared residuals is minimized by means of an alternating least squares (ALS) algorithm (more details can be found in De Roover et al., 2013b). A multistart procedure is used to reduce the probability of ending up in a local minimum.

### Model selection

When applying clusterwise SCA-P analysis, the number of clusters *C* and components *Q* need to be specified by the user. In the context of measurement invariance analysis, the number of components *Q* is equal to the number of latent variables under study, but the most appropriate number of clusters is usually unknown. To deal with this model selection problem, clusterwise SCA-P solutions are estimated using 1 to *C*^max^ clusters. Next, a scree test (Cattell, 1966) is performed to determine the number of clusters after which the increase in fit levels off: *C*^best^. Specifically, *C*^best^ is the *C*-value that maximizes the following scree ratio *sr*_(*C*)_ (see also Ceulemans and Kiers, 2006, 2009):

(4) $$sr_{\left(C\right)}=\frac{{\text{VAF}}_{C}-{\text{VAF}}_{C\;-\;1}}{{\text{VAF}}_{C\;+\;1}-{\text{VAF}}_{C}}$$

where VAF_*C*_ is the percentage of variance-accounted-for of a solution with *C* clusters (and *Q* components; for software to perform the scree test; see Wilderjans et al., 2013). VAF_*C*_ is calculated as the fitted sum of squares divided by the total sum of squares:

(5) $${\text{VAF}}_{C}=100\times \frac{\sum_{k\;=\;1}^{K}\sum_{c\;=\;1}^{C}p_{kc}{\left\|F_{k}B^{\left(c\right)}\right\|}^{2}}{\sum_{k\;=\;1}^{K}{\left\|X_{k}\right\|}^{2}}$$

Of course, differences in VAF_*C*_-values may be very small when the data contain only a few non-invariant items. Therefore, when in doubt about the optimal number of clusters, it is advised to perform the detection procedure (see below) using different *C*-values to examine the stability of the obtained set of non-invariant items, taking into account that the higher the *C*-value, the larger the number of non-invariant items may become.

### Detection of non-invariant items

To detect non-invariant items, we propose to apply the following procedure^5^, which consists of four steps:

1. Rotate cluster-specific loadings toward the postulated factor structure: Since clusterwise SCA-P solutions have rotational freedom (see above), the comparability of the cluster-specific component loadings is optimized by orthogonally rotating them toward a target matrix that corresponds to the factor model specification that was used in the measurement invariance testing (taking loadings equal to one if an item is assumed to load on a factor and zero otherwise).
2. Screen for the presence of non-invariant items: Calculate, for each cluster pair and for *q* = 1, …, *Q*, the Tucker's congruence coefficient φ (Tucker, 1951) between the *q*th cluster-specific components. The congruence coefficient is an index of similarity between components (or factors). It takes values between −1 and 1, where a negative value indicates that one of the components should be reflected, a value of zero indicates no agreement, a value between 0.85 and 0.95 indicates high similarity, and a value higher than 0.95 corresponds to virtual identity (Lorenzo-Seva and ten Berge, 2006). Therefore, in what follows, we will assume that components are identical if the congruence value is 0.96 or larger. Next, the minimal φ-value φ_min_ across these *C*(*C* − 1)/2 × *Q* congruence coefficients is calculated. When φ_min_ is less than 0.96, this suggests that the data contain non-invariant items and the procedure continues. When φ_min_ is 0.96 or larger, there is no indication that non-invariant items are present. Thus, the procedure is stopped and it is concluded that the clusterwise SCA-P analysis endorses weak measurement invariance. Note that the congruence coefficient measures the proportionality of two sets of component loadings and is thus insensitive to differences in component scale (which influence the loading sizes due to the restrictions on the component variances).
3. Detect which items are non-invariant: Remove each item one by one (i.e., with replacement) from the loading matrices and recompute the minimum congruence coefficient φ_min_ (across all cluster pairs and components), re-rotating the remaining loadings toward the corresponding subset of the target matrix. The item for which the absolute value of this φ_min_ is the highest (which indicates that the between-cluster congruence of the components improves the most when omitting this item) is considered non-invariant and permanently removed. This step is repeated until the resulting φ_min_ value exceeds 0.96, indicating weak invariance.
4. Re-estimate the cluster-specific SCA-P models for the remaining subset of items and repeat steps 1–3 to check whether additional non-invariant items are found. Continue until no more non-invariant items seem to be present (i.e., φ_min_ > 0.96). Note that the clustering is fixed in this step. Allowing an update of the clustering would often lead to a different, non-sensical clustering, because the removal of non-invariant items diminishes the differences driving the initial clustering.

This procedure differs in three important respects from the CFA procedures that were discussed in the introduction: firstly, our procedure examines the non-invariance of complete items, whereas the sequential model modification procedure and item-level invariance testing focus on the non-invariance of each loading separately. Secondly, whereas the CFA tests examine either configural or weak invariance, the procedure proposed above captures both simultaneously. Thirdly, clusterwise SCA is more parsimonious than the three CFA procedures in that it examines differences between clusters of groups rather than between separate groups, which possibly lowers the capitalization on chance.

## Applications

### Data description

In this section, we will illustrate our method for detecting non-invariant items by means of data that were originally collected to investigate emotional acculturation. Emotional acculturation refers to the process by which immigrants' patterns of emotional experience assimilate to those of the host culture (De Leersnyder et al., 2011). To investigate the robustness of the phenomenon, the researchers examined two different host cultures, and included minority groups from different heritage cultures (the cultures from which the immigrants stem). Moreover, to compare the emotional patterns of the immigrants with those of their heritage culture, two heritage groups were inspected as well (see Table 1 for an overview of the groups involved).

**Table 1:** **The 13 cultural groups under consideration and associated host country, design and sample size (note: each situation-subject combination counts as one observation)**.

**Cultural group**	**Host country**	**Design**	**Removed observations due to missing data**	**Retained observations (*N_i_***)	**Partition**
European Americans 1	USA	1	12	120	1
Korean immigrants	USA	1	21	126	1
Mexican immigrants	USA	1	16	188	1
East-Asian immigrants	USA	2	5	159	1
Latino immigrants	USA	2	1	142	1
European Americans 2	USA	2	10	122	1
Koreans	Korea	2	22	298	1
Flemish students 1	Belgium	3	5	183	2
Flemish students 2	Belgium	3	20	516	2
Belgian community	Belgium	3	26	166	2
Turkish 2nd generation immigrants	Belgium	3	17	157	2
Turkish 1st generation immigrants	Belgium	3	22	143	3
Turkish students	Turkey	3	119	699	3

*The last column indicates to which cluster the cultural group is assigned in the clusterwise SCA-P model with three clusters and two components per cluster*.

First, as previous research found emotional differences between independent and interdependent cultural contexts (e.g., Mesquita, 2001; Kitayama et al., 2006), the host and heritage cultures under study differ along the independent-interdependent dimension, with both host cultures (European American and Belgian contexts) on the independent end and all heritage cultures (Korea/East Asia, Mexico/Latino, and Turkey) on the interdependent end. A second reason for focusing on these host and heritage cultures is that they differ considerably from an acculturation point of view. The US and Belgian cultural contexts have different migration histories that translate in different policies and different collective ideas on immigrants and immigration (Van Acker, 2012). Within the US context, Korean/East Asian minorities differ from Mexican/Latino minorities in terms of both education and employment; the former are highly educated, and work white collar jobs, whereas the latter are typically less educated, and occupy blue collar jobs. Within the Belgian context, Turkish minorities tend to have little education and occupy more working class (as opposed to middle class) jobs than majority members. One of the Belgian majority samples was matched with respect to education and socio-economic status to the Turkish minority sample; the other two Belgian majority samples consisted of Belgian (Flemish) university students.

The participants reported on one to four specific situations that differed on the dimensions of valence (positive, negative), social engagement (socially engaged, socially disengaged), and social context (with friends, at home/with family, at school/work). They then rated on a 7-point Likert scale to what extent they experienced each of 17 different emotions (see **Table 3**). The situations were chosen according to three types of design. In Design 1, participants received three emotional prompts that pertained to the same type of emotional situation (e.g., positive disengaging situation), but that differed with respect to social context. In Design 2, participants received four emotional prompts that pertained to the same social context (e.g., family), but that differed with respect to type of emotional situation (i.e., positive disengaging situation, positive engaging situation, negative disengaging situation, negative engaging situation). Design 3 was similar to Design 2, but due to time constraints, participants only completed two types of emotional prompts for the same social context. The design was fixed within each group (see Table 1), which implies that differences between cultural groups may have been confounded with differences in design. Note that we removed observations (i.e., subject-situation combinations) with missing data from the data set (see Table 1).

Of course, the fact that the data contain up to four observations per subject may introduce some dependencies among the observations within a group, violating the independence assumption of the CFA framework. Retaining only one observation per subject would drastically reduce the sample size per group, leading to convergence problems when performing (multigroup) CFA analyses. However, given that for the majority of the subjects only one or two observations are included in the data (i.e., 289 subjects with one observation and 819 subjects with two observations) and that varying the type and context of the emotional situations causes substantial within-subject differences, we deem the degree of dependence in the data to be limited and not prohibitive for using the current data as an illustration for our proposed procedure.

The questionnaires (i.e., the prompts) were developed in English and then translated from English into Korean, Spanish, Dutch and Turkish, and then back-translated into English by bilingual researchers. In this pragmatic type of translation (Brislin, 1980), the accuracy of meaning is emphasized, rather than a literal, word-for-word translation.

### Configural and weak invariance testing

A latent variable structure that seems reasonable for this data set is one with a positive emotions factor and a negative emotions one (Kuppens et al., 2006). Therefore, we tested the configural and weak invariance of this latent variable structure by means of the R packages Lavaan 0.5–15 (Rosseel, 2012) and SemTools 0.4–0). To take the ordinal nature of the Likert scale ratings into account, we used the diagonally weighted least squares (DWLS) estimator (Jöreskog and Sörbom, 1996, pp. 23–24). Table 2 contains the comparative fit indices (CFI) for the CFA model for each group separately, as well as for a multigroup CFA model without imposing further equality restrictions (to evaluate configural invariance) and a multigroup CFA model with equal loadings for all groups (to evaluate weak invariance). We focused on the CFI because it is a fit index that also performs well in small samples (Hu and Bentler, 1999), which is an advantage considering the small sample size for some of the cultural groups. A CFI value of 0.95 suggests a good fit of the model to the data (Hu and Bentler, 1999), a CFI between 0.90 and 0.95 corresponds to a reasonable fit (Bentler, 1990; Tabachnick and Fidell, 2005), and a CFI value lower than 0.90 indicates a bad fit (Bentler, 1990).

**Table 2:** **Comparative fit indices (CFI) for multigroup CFA analyses imposing positive affect and negative affect factors for the emotional acculturation data**.

	**All 17 emotions**	**Seven non-invariant emotions removed**
**GROUP-SPECIFIC FIT**		
European Americans 1	**0.91**	0.99
Korean immigrants	**0.87**	0.97
Mexican immigrants	**0.81**	**0.90**
East-Asian immigrants	**0.93**	1.00
Latino immigrants	**0.89**	0.97
European Americans 2	0.97	1.00
Koreans	0.96	0.99
Flemish students 1	0.97	0.99
Flemish students 2	0.95	0.99
Belgian community	**0.94**	0.99
Turkish 2nd generation immigrants	0.97	1.00
Turkish 1st generation immigrants	0.98	1.00
Turkish students	0.97	0.98
**OVERALL FIT**		
Multigroup CFA	0.95	0.98
Multigroup CFA with equal loadings across groups	**0.86**	0.96

*CFI values lower than 0.95 are in bold face*.

First, we examined configural invariance by looking at the CFI value of the unconstrained multigroup model. The CFI value is 0.95; thus, at first sight the baseline model with the positive and negative affect factors seemed to be appropriate (i.e., configural invariance confirmed). However, the CFI values for the separate groups conveyed that this model had an excellent fit for some groups but not for all, with CFI < 0.90 for the Korean, Mexican, and Latino immigrants.

Second, we looked at the overall fit of the weak invariance model (i.e., equal loadings across all groups). The CFI value of 0.86, and also the difference of 0.09 in CFI with the overall configural invariance model, indicated a bad fit of the model to the data and, thus, a flat out rejection of weak invariance.

### Clusterwise SCA and the detection of non-invariant items

**Figure 1:**
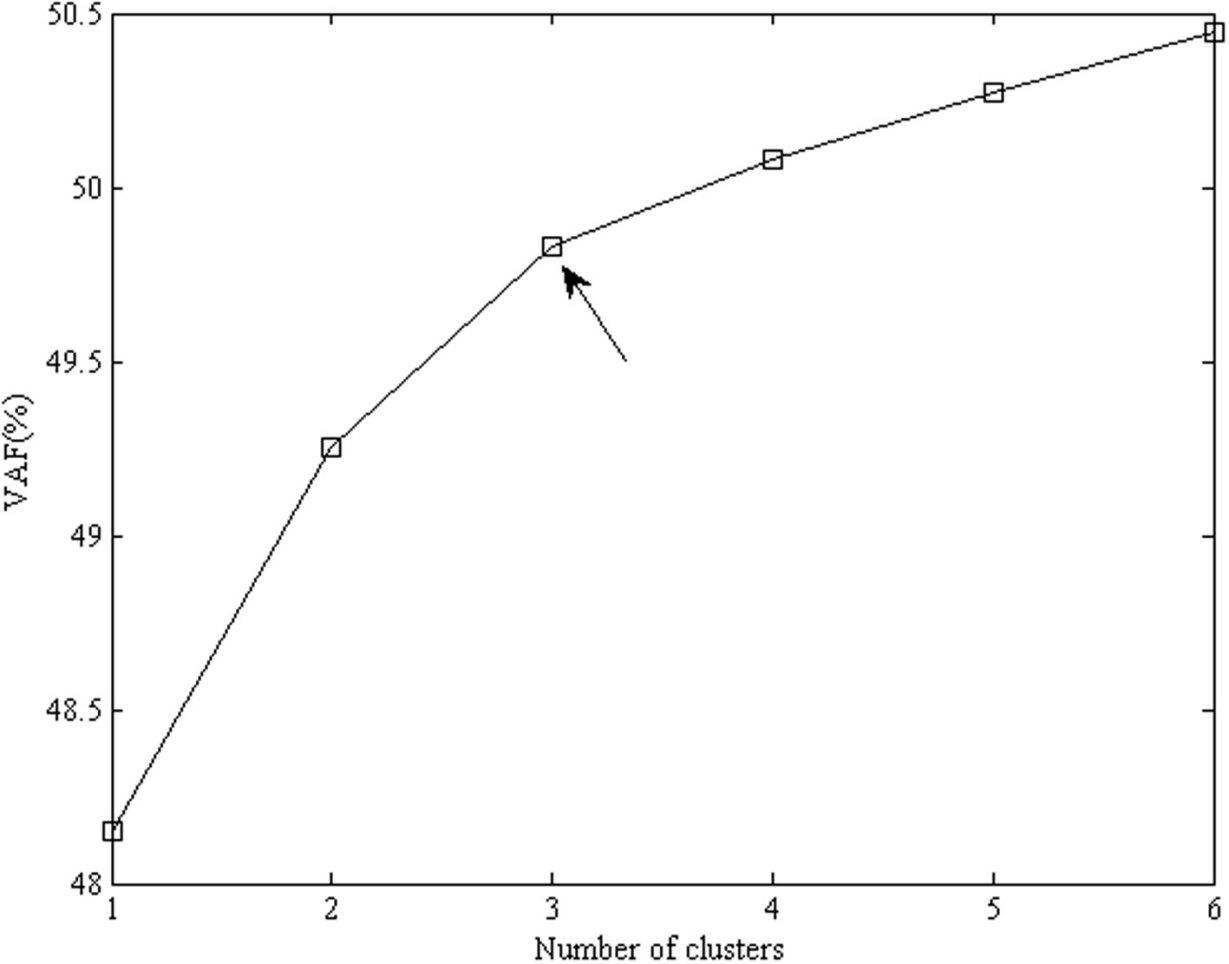
**Percentage of explained variance for clusterwise SCA-P solutions for the emotional acculturation data, with the number of components equal to 2 and the number of clusters varying from 1 to 6**. The favored number of clusters is 3 (indicated by the arrow), because the increase in fit levels off after three clusters.

To investigate whether the lack of invariance is due to the presence of non-invariant items, we centered the data per group and normalized them over groups and applied clusterwise SCA-P analyses with 1–6 clusters and two components per cluster. A scree plot with the VAF values of the resulting models is presented in Figure 1. Although fit differences are small, the increase in fit clearly levels off after three clusters. This is also confirmed by the scree ratio's, which amount to 1.9, 2.3, 1.3, and 1.1 for two, three, four, and five clusters, respectively. Thus, we proceeded with the clusterwise SCA-P model with three clusters and two components per cluster.

The corresponding partition of the cultural groups is shown in Table 1. The cultural groups living in the USA are gathered in Cluster 1, together with the Koreans. Cluster 2 consists of the indigenous Belgian groups, together with the second generation Turkish immigrants in Belgium. Cluster 3 contains the Turkish students living in Turkey and the first generation Turkish immigrants in Belgium. The fact that the second generation Turkish immigrants were assigned to the Belgian cluster suggests that these immigrants acculturated with respect to the meaning of their emotions. The assignment of the Korean immigrants as well as the Koreans to the USA cluster, indicates that—in case of three clusters and two components—neither of them stands out enough in terms of their covariance structure to end up in a separate cluster. Note that all the data in cluster 1 were gathered by means of Designs 1 and 2, whereas the data in clusters 2 and 3 were collected using Design 3 only. Thus, it is possible to clearly interpret the differences between cluster 2 and 3 as differences in cultural groups, whereas the differences between cluster 1 on the one hand and clusters 2 and 3 on the other, may be due to design differences, in addition to cultural differences.

The target (i.e., positive emotions component and negative emotions component) rotated loadings of the three clusters are given in Table 3. At first sight, the component structure in all three clusters closely resembles this target structure: a first component that mainly corresponds to the positive emotions and a second component that is mainly constituted by negative emotions. Similarity to the target structure was corroborated by the congruence values between the cluster-specific components and the corresponding columns of the target structure, which always exceeded 0.85 indicating high similarity—but not identity—to the target structure (see Table 4).

**Table 3:** **Cluster-specific loadings for the clusterwise SCA-P model with three clusters and two components per cluster, orthogonally Procrustes rotated toward a positive and negative target structure**.

**Emotions**	**Cluster 1 (USA and Koreans)**		**Cluster 2 (Belgian)**		**Cluster 3 (Turkish)**	
	**Pos**.	**Neg**.	**Pos**.	**Neg**.	**Pos**.	**Neg**.
Respect	**0.76**	−0.18	**0.72**	−0.20	**0.86**	−0.19
Interested	**0.69**	−0.24	**0.63**	−0.26	**0.69**	−0.11
Helpful	**0.75**	−0.17	**0.63**	−0.12	**0.61**	−0.14
Close	**0.64**	−0.23	**0.74**	−0.02	**0.79**	−0.24
*Strong*	***0.66***	*−0.28*	***0.47***	***−0.46***	***0.75***	*−0.28*
*Proud about myself*	***0.64***	***−0.43***	***0.49***	***−0.58***	***0.68***	*−0.34*
*Relying*	***0.53***	*0.30*	***0.76***	*−0.01*	***0.78***	*−0.09*
*Surprised*	*0.26*	*0.30*	*0.31*	*−0.12*	***0.72***	*−0.13*
Ill feelings	−0.35	**0.51**	−0.39	**0.55**	−0.39	**0.69**
Upset	−0.35	**0.74**	−0.37	**0.62**	**−0.47**	**0.69**
Irritated	−0.25	**0.69**	**−0.57**	**0.54**	−0.20	**0.67**
Embarrassed	−0.12	**0.79**	−0.18	**0.60**	−0.18	**0.73**
Ashamed	−0.09	**0.81**	−0.19	**0.77**	−0.16	**0.65**
Guilty	−0.22	**0.73**	−0.14	**0.82**	−0.26	**0.69**
*Bored*	*0.07*	***0.40***	*−0.25*	***0.35***	***−0.51***	***0.74***
*Indebted*	***0.45***	***0.42***	*0.27*	***0.74***	*0.27*	***0.53***
*Resigned*	*−0.08*	***0.60***	*0.06*	*0.32*	***0.40***	*0.33*

*Loadings larger than 0.40 in absolute value are indicated in bold face. Non-invariant items are indicated in italic*.

**Table 4:** **Tucker's congruence coefficients between the cluster-specific component loadings in Table 3 and the target structure (per component), as well as between the cluster-specific components mutually (per component and per cluster pair), when including all variables**.

		**Cluster 2**		**Cluster 3**		**Target structure**	
		**Positive**	**Negative**	**Positive**	**Negative**	**Positive**	**Negative**
**Cluster 1**	**Positive**	0.94	-	0.90	-	0.89	-
	**Negative**	-	0.93	-	0.93	-	0.90
**Cluster 2**	**Positive**			0.94	-	0.86	-
	**Negative**			-	0.95	-	0.88
**Cluster 3**	**Positive**					0.89	-
	**Negative**					-	0.94

However, we did notice some remarkable between-cluster differences for specific items. For instance, “surprised” has a high loading on the “positive” component in the Turkish cluster and a moderately high positive loading on the “negative” component in the USA cluster. These differences were confirmed by the Tucker's congruence coefficients between the corresponding cluster-specific components (see Table 4), which lay between 0.90 and 0.95, indicating between-cluster differences in loading structure.

Applying the procedure described in the Methods section, yielded the following seven non-invariant items: “strong,” “proud about myself,” “surprised,” “relying,” “resigned,” “bored,” and “indebted.” After removing the seven non-invariant items and estimating a new SCA-P model per cluster for the retained subset of variables, the congruence coefficients of the components between clusters ranged from 0.96 to 0.99. Against the background of other research on the cultures of comparison, it is possible to meaningfully interpret some of these non-invariant items. For instance, “proud about myself” has a higher negative loading on the “negative” component in the Belgian cluster. This indicates that when Belgians experience negative emotions, they feel less proud about themselves than people belonging to the other cultural groups. The association between negative emotions and feeling less proud is also, to a lesser extent, observed in the USA and Koreans cluster. The different meaning of “proud about myself” between the Turkish cluster on the one hand, and the Belgian and USA and Koreans cluster may be understood in the light of the specific meaning that this concept takes on in cultures that emphasize “independence” (e.g., Markus and Kitayama, 1991): in these cultures, pride has the connotation of being successful and superior (Roseman, 2013), and thus may be seen as compromised by failure which is associated with negative emotions.

As another example, “relying” has a moderately high positive loading on the “negative” component in the USA and Koreans cluster. Follow-up analyses showed that the negative connotation of “relying” in this cluster is mainly driven by the clear negative connotation among the European Americans (in an SCA-P model for the two groups of European Americans “relying” had a loading of 0.42 on the negative component), which is less outspoken in the USA immigrant groups (loading of 0.27) and among Korean natives (loading of 0.24). The feeling of relying on someone else may have a negative connotation (and co-occur with negative emotions) for the European Americans, because it clashes with central ideals of personal autonomy and self-reliance (e.g., Markus and Kitayama, 1991).

Another interesting difference is the fact that “resigned” has a lower loading on the “negative” component in the Belgian and Turkish clusters in comparison to the USA and Koreans cluster. Moreover, in the Turkish cluster, “resigned” loads primarily on the “positive” component. The different meanings of “resigned” may be associated with different ideas on control. Personal control is a central value in middle class American culture, where it is considered instrumental to an individual's independence and autonomy (Markus and Kitayama, 1991); in this context, resignation is likely to have the negative connotation of giving up. On the other end of the spectrum, Turkish culture emphasizes “kismet” or fate: Turkish people tend to have a strong belief in both fate (e.g., Ergüder et al., 1991) and authority (Dağ, 1991; Lester et al., 1991). Therefore, feeling resigned may be regarded as positive in the Turkish culture, as it denotes that one accepts an event and one's fate.

To summarize, important differences in component structure were found, indicating that a subset of the emotions covary differently with the other emotions or are even valued differently in some of the cultural groups. Surely, these cross-cultural differences are interesting in itself. Furthermore, these differences may be what's hampering the measurement invariance testing, as they pertain to both the primary (e.g., “surprised” being less strongly associated with the “positive” component in the USA and Turkish clusters) and secondary loadings (e.g., “resigned” being part of positive affect in the Turkish cluster), which may, respectively, explain the rejection of the weak invariance model and the bad fit of the configural invariance model for some of the groups.

### Modified configural and weak invariance testing

To examine whether the detected non-invariant items were indeed contributing to the violations of measurement invariance, we removed these seven items from the data and re-evaluated the configural and weak invariance CFA models mentioned above. Regarding configural invariance, the resulting CFI values for the separate groups were higher (see Table 2), leaving only one group (i.e., Mexican immigrants) in the reasonable fit range (i.e., CFI between 0.90 and 0.95) and none in the bad fit range (i.e., CFI < 0.90). The same holds for the CFI value of the multigroup model, which amounted to 0.98 and suggested an excellent overall fit. Regarding weak measurement invariance, the corresponding CFA model had a good CFI of 0.96, as compared to 0.86 when all items were included. Surely, the fit decrease of 0.02 when going from the configural invariance model to the weak invariance model (i.e., from 0.98 to 0.96) was still large enough to reject weak invariance, but clearly our procedure pinpointed some interesting differences in emotion covariances that were interfering with weak invariance.

Another strategy for incorporating the results of our procedure in the CFA testing is freeing some of the loadings of the non-invariant items. Regarding configural invariance, we added secondary loadings (instead of zero ones) for the non-invariant items. The overall CFI for the resulting multigroup CFA model was 0.98, whereas the group-specific fit values were very similar to those in Table 2. Regarding weak invariance, allowing both loadings of the non-invariant items to vary across groups yielded a partial weak invariance model with a CFI value of 0.96.

### Results of CFA methods for dealing with invariance violations

To compare our results to those of popular CFA methods for dealing with invariance violations, we applied the three procedures discussed in the Introduction. In the sequential modification procedure (MacCallum et al., 1992; Stuive et al., 2009), we confined ourselves to modifying the weak invariance model by allowing primary loadings to differ in certain groups or adding secondary loadings for certain groups, because several authors have reported that this modification procedure outperforms methods which allow for other modifications (e.g., including residual covariances; MacCallum, 1986; Silvia and MacCallum, 1988). We continued freeing or adding loadings for specific groups, as specified by the modification indices, until the resulting increase in fit (ΔCFI) no longer exceeded 0.01. As a result, the primary loading of “bored” was freed for group 4 and a secondary and free loading was added for “resigned,” also for group 4. The CFI of the resulting partial weak invariance model is 0.86.

The item-level invariance testing (Cheung and Rensvold, 1999) entailed no less than 66 additional invariance tests (see Introduction); i.e., two factor-specific tests, 28 tests for the non-zero loadings on the positive factor and 36 tests for the non-zero loadings on the negative factor. The integrated results of these tests indicate that the primary loadings of “surprised,” “relying,” “resigned,” “bored,” and “helpful” have to be freed across the groups. The CFI of the thus obtained partial weak invariance model is 0.92.

The strategy presented by Byrne and van de Vijver (2010) involved two times 17 additional multigroup CFA analyses; i.e., deleting one item at a time, for configural invariance on the one hand and for weak invariance on the other hand. With respect to configural invariance, only one item yielded a CFI increase of more than 0.01 upon deletion: “indebted.” Thus, for “indebted,” there seemed to be some misfit with respect to the imposed factor structure, possibly due to the need for a secondary loading of indebted on the positive component for some of the groups. Deleting “indebted” led to an overall CFI of 0.97 and group-specific fit values ranging from 0.85 to 0.99 with only the Mexican immigrants having a CFI below 0.90 (i.e., 0.85, implying bad fit). With respect to items “interested”, “helpful”, “close”, “relying”, “ill feelings”, “embarrassed”, and “ashamed”, no decision could be made, since the corresponding multigroup CFA analyses (i.e., with one of these items being deleted) did not converge. With respect to weak invariance, five non-invariant items were traced by this approach: “surprised,” “relying,” “resigned,” “bored,” and “indebted.” When deleting this subset of items the overall CFI of the multigroup CFA with equal loadings across groups amounted to 0.96.

### Conclusion with respect to the cross-cultural emotion data

This application demonstrated that using clusterwise SCA to investigate what is causing a lack of measurement invariance makes sense, because (1) the fit of the multigroup CFA models improved greatly when the detected non-invariant items were removed or when the models were modified by allowing for specific secondary loadings or by letting particular primary loadings vary across the groups, and (2) the detected set of non-invariant items largely overlapped with those resulting from the three multigroup CFA procedures. Also, the unique aspect of the proposed approach—the clustering of the groups—was nicely illustrated, i.e., meaningful clusters of groups were found and the non-invariant items could be traced by comparing the loadings between these clusters, without having to inspect the loadings of each group separately. Moreover, the total CPU time of the clusterwise SCA-P analyses, i.e., including the model selection and the detection procedure was about 33 s only (using Matlab R2013b on an Intel® Core™ i7-3770K processor of a personal computer, with a clock frequency of 3.4–3.9 GHz and a RAM speed of 1600 MHz) while the item-level invariance testing and the Byrne and van de Vijver (2010) approach were much more cumbersome and time-consuming (on the same computer, the former procedure took more than 24 h to run and the latter about 2 h and a half, using the R-packages Lavaan 0.5–15 and SemTools 0.4–0). Applying the sequential model modification procedure took only 8 min, but this was because it led to only two modifications with a ΔCFI > 0.01 (and, consequently, did not improve the model fit very much).

## General discussion

The issue of measurement invariance is ubiquitous in the behavioral sciences nowadays as more and more studies yield multivariate multigroup data. Although CFA based methods have been proposed to trace which items are hampering measurement invariance, these methods have some disadvantages in that they require researchers to run a multitude of analyses and in that they imply assumptions that are often questionable. In this paper, we proposed an alternative strategy which consists of running clusterwise SCA and comparing the resulting loadings via congruence coefficients to quickly trace possible non-invariant items. The cross-cultural application demonstrated that this novel approach is useful and can co-exist with the traditional CFA approaches.

As also holds for the discussed CFA approaches, it may sometimes occur that invariance is still rejected after removing the items indicated as non-invariant by the new approach. In such cases, one may consider the following actions to further pursue invariance.

Firstly, it may be that the number of clusters was too small to detect all non-invariant items. Thus, it may be useful to examine a clusterwise SCA solution with more clusters—for the complete set of items—and repeat the detection heuristic.

Secondly, when the overall fit of the baseline multigroup CFA model is still bad, this suggests that the CFA model is misspecified. For example, additional factors may be needed to approach a good fit, the postulated latent variable model may be completely off or distributional assumptions may be violated. If so, the clusterwise SCA based detection approach will not be able to remedy this problem and neither can the CFA approaches. To get more grip on what is going on, exploratory factor analysis may be used to examine the factor structure. Moreover, problems with regard to the target structure can be easily traced from the clusterwise SCA results by checking whether the congruence coefficients between the cluster-specific components and the postulated factors are low.

Thirdly, when the fit of the baseline CFA model remains below standards for only one or a few of the groups after removing the detected non-invariant items, it may be that the group(s) in question need other CFA model modifications such as residual covariances. To this end, one may resort to the group-specific modification indices.

Fourthly, when configural invariance is established but weak invariance is still rejected, a more strict congruence criterion may be needed for the data at hand (e.g., 0.97 instead of 0.96) to detect all subtle size differences in loadings which may be causing the rejection of weak invariance. Especially when the number of invariant items is much larger than the number of non-invariant items, it may happen that the congruence criterion is not strict enough to detect the most subtle differences.

Fifthly, it may be the case that the factor structure is appropriate for most groups but incorrect for a minority of outlying groups. Clusterwise SCA will conveniently assign these outlying groups to one or more separate clusters, with the congruence coefficients between the corresponding cluster-specific and the a priori factor structure being low. For such data, one may want to remove the outlying groups and repeat the measurement invariance testing. In this regard, Byrne and van de Vijver (2010) specified a set of criteria to identify groups that are possibly outlying in terms of their item scores and evaluated the goodness-of-fit of the multigroup CFA model when deleting these groups one by one (i.e., with replacement). However, these criteria are based on the level of the items^6^ rather than on their factor structure. This implies that this approach is not ideal to track groups with outlying factor structures.

Finally, it may be that measurement invariance simply cannot be established because the groups form a few clusters that are characterized by a distinct factor structure. Using clusterwise SCA, one can conveniently discern such clusters and perform the measurement invariance testing within the clusters. Since, up to now, no factor analytic counterpart exists, clusterwise SCA is the only method to find clusters of groups based on within-group component or factor structure without having to resort to tedious pairwise comparisons of group-specific structures.

As a final remark, an attractive feature of the proposed approach is that its applicability, unlike the CFA based methods, largely surpasses the context of measurement invariance. Indeed, the approach can also be used when researchers do not have an a priori idea about the underlying structure of the items and about possible differences across groups (e.g., Krysinska et al., in press). To this end, a standard SCA-P analysis (i.e., without clustering) is run on the data and the resulting component loadings—the “common component structure”—are used as the target structure toward which the cluster-specific loadings are rotated.

## Author notes

Kim De Roover is a post-doctoral fellow of the Fund for Scientific Research Flanders (Belgium). The research leading to the results reported in this paper was sponsored in part by Belgian Federal Science Policy within the framework of the Interuniversity Attraction Poles program (IAP/P7/06), and by the Research Council of KU Leuven (GOA/2010/02).

### Conflict of interest statement

The authors declare that the research was conducted in the absence of any commercial or financial relationships that could be construed as a potential conflict of interest.

## Footnotes

^1^Although the theoretical comparability of component and factor analyses has been heavily debated (e.g., Gorsuch, 1990), Velicer and Jackson (1990) established that component and factor loadings usually are highly similar and lead to the same interpretations with respect to underlying constructs.

^2^In this paper we focus on the frequentist framework when discussing different methods to investigate the lack of invariance. Note that also in the Bayesian framework, methods exist with a similar aim (e.g., Muthén and Asparouhov, 2012, 2013).

^3^The item for which the factor loading is fixed to one in each population for model identification.

^4^One may argue that this advice is somewhat inconsistent with the fact that measurement invariance testing is usually done on raw data. Multigroup CFA is less sensitive to between-item differences in variability, however, due to the many restrictions on the factor loadings (i.e., zero or equality constraints). Moreover, if the variance differences are relatively small, analyzing unrescaled data (i.e., data that is only within-population centered) will yield very similar results to those obtained when overall rescaling is performed. For instance, for our illustrative data set (see Application section), the clustering of the groups was identical and the subset of non-invariant items consisted of the items reported below with the exception of “proud about myself.” The CFI indices of the multigroup CFA models that are obtained when these six items are removed, are almost identical to the reported ones.

^5^The procedure as well as the clusterwise SCA-P analyses are implemented in a Matlab R2013b function which can be obtained freely from the first author.

^6^Note that a convenient approach for identifying countries that are deviant with respect to item level was proposed by Ceulemans et al. (2013). This approach was based on robust principal component analysis.
